# Powdered Soap as a Cause of Death among Swill-fed Hogs1Bulletin Cornell University Agricultural Exepriment Station.

**Published:** 1898-05

**Authors:** Veranus A. Moore

**Affiliations:** New York State Veterinary College, Ithaca, N. Y.


### POWDERED SOAP AS A CAUSE OF DEATH AMONG SWILL-FED HOGS.[1]

By Veranus A. Moore, B.S., M.D.,
NEW YORK STATE VETERINARY COLLEGE, ITHACA, N. Y.

It is a common experience of those who are engaged in the investigation of animal diseases to occasionally find outbreaks of a peculiar nature among swill-fed hogs. By these are meant herds of greater or less size, usually kept near or within the outskirts of our villages or small cities, and which are fed upon the kitchen refuse, often including the dishwater, collected from hotels, boarding-houses, and private dwellings. The cause of death in these outbreaks is, in this State, at least, usually attributed to hog-cholera. The basis for this popular diagnosis seems to be in the similarity of certain of the symptoms manifested by these animals to those of hog-cholera, such, for example, as diarrhœa and partial paralysis, and the fact that a majority of those attacked die. The

[1] Bulletin Cornell University Agricultural Exepriment Station.

course of the disease is irregular, deaths occurring in from a few hours to several days after the symptoms appear.

During the past year I have had occasion to make investigations into the nature of several of these outbreaks of a supposedly infectious disease. In a few of these epizoötics hog-cholera or swine-plague was easily demonstrated. In certain others, however, these or other infectious diseases could not be found. The animals were usually fed the kitchen slops collected from hotels and boarding-houses. The tissue changes in the animals examined were typical of no known disease, and notwithstanding the bacteriological examinations which were made, together with animal inoculations with pieces of the diseased organs, the cause of death remained undetermined. The post-mortem examinations showed in nearly all of these animals enlarged and dark-colored lymphatic glands, especially those of the mesentery. The bloodvessels of the mesentery were very much distended with blood. The liver and kidneys were usually not affected, but occasionally these organs were involved. Where there had been marked nervous symptoms the brain was much congested. Occasionally the lungs contained areas of collapse. As a rule, the intestines were pale, and the mucous membrane seemed to be abnormally shiny.

The negative outcome of these investigations suggested that possibly our methods had been faulty or that some unknown conditions existed which had obscured the cause of death, and that, after all, the popular diagnosis of an infectious disease was right. Against this theory was the fact that the disease did not spread from the affected herds to others, although, as a rule, precautions were not taken to prevent its dissemination, and in some instances the neighboring herds were most favorably situated for contracting the disease if it had been contagious. In certain of the outbreaks the exceedingly filthy condition in which the pens and yards were kept suggested, in the absence of a knowledge of definite specific agents, that the animals had died as a result of their unsanitary surroundings and unwholesome food, a hypothesis which in some instances is still entertained as being highly probable. However, we were still confronted with the problem that in many outbreaks neither a specific infectious disease could be found nor the exciting cause of death pointed out.

Although it was apparent that the cause of the deaths was to be found in the food, the feeders of this kind of swill failed to see why they should discontinue its use. Naturally they felt that if we could not find or demonstrate the presence of the destructive agent in the swill the cause of death must be something else, probably hog-cholera, for thousands of hogs are annually raised upon this kind of food. Further, the plea that such garbage was not a suitable or even wholesome food for their animals availed nothing, for the reply was that usually their pigs thrived upon it.

Early in the summer, in conversation on this subject with Mr. W. F. Davey, an enterprising farmer living near Brewerton, N. Y., he related the circumstances concerning an outbreak of this kind in which he had traced the cause of the trouble to the soap used in washing the dishes. The swill, including the dishwater, was collected from three small hotels and fed to a herd of swine. In a short time the animals began to sicken, and many of them died. Upon inquiry it was found that in the hotels large quantities of powdered soap were used in washing the dishes. This was stopped, and no more animals died. Later in the season Dr. J. A. McCrank, of Plattsburg, told me of an outbreak of an apparently infectious disease among swine which had come under his observation, and in which he could not make a positive diagnosis. In the investigation of its cause he found that the hogs were being fed the swill, including the dishwater, from a hotel. Upon inquiry he found that powdered soap was being used in large quantities. The swill from this place was stopped, and the disease disappeared.

In following up the line of inquiry which these experiences suggested, it was found that there is among the more enterprising farmers a quite general belief that these soaps, when given in considerable quantities, are injurious and even fatal to hogs. The consensus of opinion on this subject, together with the more definite observations of Mr. Davey and Dr. McCrank, appeared to be so conclusive that it seemed important to determine by careful experiment to what extent, if at all, powdered soaps can be considered as the cause of death in this class of outbreaks. To this end the experiment about to be described was carried out. It shows that when certain of the powdered soaps sold in the market are present in the food in relatively large quantities a considerable percentage of the animals will sicken and many of them will die.

When, however, the soaps are added to the food in small quantities (a dessertspoonful in the food for three pigs, twice daily) no bad effects seem to follow. The cause of death when it does occur is probably due, as shown by the chemical analyses, to the free alkali, sodium carbonate, or washing-soda, which they contain.

*Experiment in Feeding Powdered Soaps to Pigs.*

In the experiment three of the commonly-used powdered soaps were selected. They are here designated as soaps A, B, and C. Nine pigs, weighing about twenty pounds each, were taken. They were given their regular food, grain mixed in water, and some separator-milk. To this was added a definite quantity of the soaps, which were dissolved and thoroughly mixed in the food twice daily.

SOAP A.—*July* 10*th*. Pigs Nos. 1, 2, and 3 were placed in pen No. 1. They were given, night and morning, regular rations as previously described, to which were added 2 ounces of soap A.

14*th*. Pigs well. Quantity of soap given increased to 4 ounces.

18*th*. Pig No. 1 has profuse diarrhœa, others well.

20*th*. Pig No. 1 has diarrhœa; at times it runs about the pen in apparently a dazed condition.

24*th*. Pigs Nos. 1 and 2 have bad diarrhœa. Quantity of soap given reduced to 1 ounce.

*August* 1*st*. Pigs appear to be well.

7*th*. Quantity of soap increased to 5 ounces.

8*th*. Pigs sick; all have diarrhœa; do not eat; have some difficulty in walking.

9*th*. Pigs appear to be no better.

11*th*. Pigs still sick; have eaten very little. Soap stopped.

12*th*. Pigs slightly better.

15*th*. The condition of the pigs is much improved.

18*th*. Animals apparently well.

The feeding of this soap was repeated on these animals some weeks later with a similar result.

SOAP B.—*July* 10*th*. Pigs Nos. 4, 5, and 6 were placed in pen No. 2. They were fed the regular rations to which were added, morning and evening, 2 ounces of soap B.

14*th*. Pig No. 4 has a bad diarrhœa; others well. Quantity of soap given increased to 4 ounces.

15*th*. All three have a diarrhœa.

19*th*. Pig No. 4 found dead. No. 5 very sick, unable to stand, refuses food.

20*th*. Pig No. 5 cannot stand, limbs constantly jerking; there seems to be paralysis; it dies late in the afternoon. Pig No. 6 has suffered from diarrhœa, but otherwise seems to be well, although it eats very little. Quantity of soap reduced to ½ ounce.

22*d*. Pig No. 6 better.

*August* 1*st.* Pig No. 6 much improved. Soap discontinued.

Soap C.—*July* 10*th.* Pigs Nos. 7, 8, and 9 were placed in pen No. 3. They were fed the same as the others. Night and morning, 2 ounces of soap C were mixed with their food.

13*th.* All the pigs have diarrhœa; eat very little.

14*th.* Quantity of soap reduced to 1 ounce.

16*th.* Pigs very sick; eat very little; head jerks constantly; limbs tremble; temperature 103.5°, 104°, 103.8° F.

18*th.* Pig No. 7 dies suddenly to-day; others still sick. Pig No. 8 has much difficulty in standing; lies with feet extended, legs and head are constantly jerking. No. 9 has diarrhœa, eats little, but otherwise appears to be well.

19*th.* Pig No. 8 found dead this morning. No. 9 seems to be better.

20*th.* Pig No. 9 eats heartily; appears to be quite well.

Three other pigs, Nos. 10, 11, and 12 from the same lot, were placed in pen No. 3 with pig No. 9. They were given ½ ounce of soap C, thoroughly mixed with their food, twice daily.

25*th.* Pigs apparently well.

*August* 1*st.* Pigs apparently well.

17*th.* Pigs apparently well. The quantity of soap increased to 4 ounces at each feeding.

18*th.* Pig No. 10 sick.

20*th.* Pigs all sick, refuse food. They ate sparingly of some corn given them.

21*st.* No appreciable change.

23*d.* Pig No. 10 very sick. The muscles of the head and legs constantly jerking. Eats very little of the regular food, but partakes sparingly of corn.

25*th.* No change.

27*th.* Pigs very sick; have refused food containing soap for two days. Eat sparingly of corn. Soap discontinued.

The pigs which recovered from the immediate effect of the soap did not become thrifty for some weeks. It was late in September before they began to show signs of growth.

*Postmortem Examination.*

Pig No. 4. The skin over the ventral part of the body and between the thighs of a pinkish color; kidneys very pale; spleen normal. The bloodvessels of the mesentery much congested, the mesenteric glands enlarged and œdematous, many of them congested. Areas of the mucous membrane of the intestines, especially the ileum, were of a dark-reddish color. The lungs and heart were not changed. The brain was deeply congested.

Pig No. 5. This pig showed lesions very similar to those exhibited by No. 4. The essential difference was an increase in the intestinal congestion.

Pig No. 7. The skin between the thighs and about the nose was of a bright pinkish color. The liver was small, exceedingly firm and friable. The mesenteric bloodvessels were injected, and the mesenteric glands were enlarged and œdematous, and many of them deeply reddened ; a few were hemorrhagic. Spleen normal. The cortex of the kidneys very pale, but the papillæ were abnormally dark. The mucous membrane of the intestines was congested in a few irregular areas, and the mucosa of the stomach covered with a thick layer of mucus. The heart and lungs were normal in appearance. The brain was very much congested.

Pig No. 8. The tissue changes in this animal were similar to those found in pig No. 7, with the exception that the kidneys were much congested.

A careful bacteriological examination was made of the liver, spleen, kidneys, and blood of each animal that died. In nearly every instance (all but two) the tubes of culture-media (agar and bouillon) inoculated remained clear. The two exceptions contained saprophytic bacteria and were probably contaminations from the air. This examination shows that the alkali had not favored the migration of the bacteria from the intestines to the various organs of the body.

In order to check the result, several pigs from the same litters as those used in the experiment were kept, in adjacent pens, and given the same kind of food. They all remained well. This fact, in addition to the negative results from the bacteriological examination, and the peculiar nature of the lesions, are sufficient evidence that the sickness and the fatalities among the pigs in the experiment were due to the soaps administered.

It is important to note that the lesions found in the pigs which died in the experiment were similar to those found in the pigs in certain of the outbreaks mentioned among swill-fed hogs. Considering the facts as they appear, it seems highly probable that the cause of death of the animals in certain of the outbreaks mentioned was the presence of the free alkali in the swill. This hypothesis is supported by the experiences of Mr. Davey and Dr. McCrank.

*Chemical Analysis of the Soap-powders Used.*

In order to ascertain the chemical nature of these soaps they were submitted to Mr. Geo. W. Cavanaugh, assistant-chemist of the Agricultural Experiment Station, for analysis. The following report was received:

"The soap-powders used in the above experiment are mixtures of ordinary hard soap, that has been powdered or in some way reduced to a fine condition, and sodium carbonate. Sodium carbonate is known in commerce as sal soda, washing-soda, or soda. In water it forms a caustic solution which is the lye used in making the old-fashioned hard soaps. Analysis: Soap A, 49.60 per cent.; soap B, 55.42 per cent.; soap C, 55.54 per cent."

A careful inquiry has been made to ascertain the quantity of these soaps commonly used in washing dishes. This has revealed the fact that while the amount used by different individuals varies, the quantity is large, usually far in excess of the amount prescribed by the manufacturers. Thus I have been told, by thoroughly reliable people, of dishwashers who would use one-third of a box in cleaning the dishes after a single meal. While this is extreme, it is said not to infrequently happen, and it is easy to understand that the swill from these kitchens would contain far more of the alkali than we found necessary to produce fatal results. Should such excess in the use of these cleaning agents be indulged in for several days in succession, we have, in the light of the foregoing experiment, a cause for many fatalities among the hogs fed upon the dishwater.

In view of this danger it seems better to abandon altogether the habit of giving dishwater to hogs. Although the feeding of garbage is generally condemned, the scraps of vegetables and table-refuse could, perhaps, if properly collected be used with safety. But certainly pure water is a much more wholesome drink, even for swine, than dirty dishwater. When the subject of "swill feeding" as a business is studied, and the conditions as they exist are understood, the wonder is not that some of the hogs die, but rather that any of them live.

It is not presumed that the poisoning by carbonate of sodium is the only cause of death among swill-fed hogs. Other destructive agencies are liable to be found in the decomposing garbage. The results of the investigation which the necessity of good farm-hygiene demands will very likely disclose the specific nature of many of them. Another fact worthy of consideration is that the investigation of the last year shows that nearly all of the outbreaks of hog-cholera and swine-plague which came to our attention started among herds of swine fed upon garbage and swill collected from the sources above mentioned. This is significant, and it points to the undesirableness of feeding garbage to animals. In fact, if the total losses it occasions are counted, it is questionable if anything is gained in this attempt to save waste-products. It is stated in the official reports of the U. S. Department of Agriculture that in 1896 12 per cent. (which amounts to 5,440,176) of the hogs in this country died from disease.

Again, it has long been recognized that the feeding of garbage to hogs furnishes one of the most favorable channels for the introduction of hog-cholera and swine-plague bacteria. As a rule, wherever we find hogs in clean, well-ventilated pens, and fed upon wholesome food, we find thrift and health, and where the animals are surrounded with disgusting filth and fed upon decomposing slops or other unwholesome food we expect to and often do find disease.

It is, unfortunately, becoming a too-prevalent habit among our farmers to assume, as soon as one or two pigs die that some infectious disease, such as hog-cholera, is among them. It is further most unfortunate that they frequently entertain the fatalistic notion that a remedy is beyond their reach. Fully 25 per cent. of the outbreaks of reputed hog-cholera which we have investigated during the past year have not been hog-cholera or any other known infectious disease. While it is true that when hog-cholera becomes well established in a herd there is great danger that the majority of the animals will die, it is equally true that if the disease is not a genuinely infectious one that a majority of the animals can, by proper treatment, be saved. When a pig sickens and dies the thing to do is to examine, or have it carefully examined, to find out, if possible, what the cause of death is, in order that the best methods known for preventing the further spread of the disease may be promptly adopted.

If the examination shows the disease to be hog-cholera, swine-plague, or any other infectious disease, like anthrax or tuberculosis, the as yet uninfected and apparently well animals should be placed in other pens and the old ones disinfected. The animals should be given easily digested and nourishing food, plenty of sunlight and pure air. If others should become affected, the well ones should again be separated from the sick. The channel or way by which the specific cause of the disease got into the herd should be diligently sought for. As the most common way is through the food, it is always a safe precaution to change the diet.

It is certainly not desirable to acquire the reputation of having an infectious disease among one's animals when the real trouble is due to poor hygiene, to some irregularity in their care, or to an accidental poisoning.

If the diagnosis cannot be positively made it is best to put the apparently well hogs in a separate pen, provide them with good ventilation, wholesome food, and cleanliness. *It is important that the food should be changed.* By carefully observing the method of *strict isolation*, disinfection, healthful surroundings, and nourishing diet, many epizootics of infectious diseases have been checked, and it is safe to presume that if such precautions were rigidly adhered to nearly all of the losses now sustained from dietary causes would be saved. The observance of the rules necessary for the promotion of good health among mankind apply with equal force to the lower animals.

*Conclusions.*

From the foregoing the following conclusions seem to be warranted :

1. The greatest amount of loss sustained from swine-diseases in this State is among hogs fed upon the swill collected from hotels, boarding-houses, and other large institutions.

2. The cause of death in certain outbreaks of disease among swill-fed hogs is the direct poisoning of the animals by the excess of free alkali (washing-soda) in the swill. These alkalies come from the powdered soaps used in washing dishes.

3. It appears that small quantities of the powdered soaps do not immediately produce bad results. It is presumable that they can be used in quantities sufficient for the needs of cleanliness with perfect safety ; but owing to the danger involved in their use it is safer not to give the water containing them to animals.

4. In addition to the unwholesomeness of garbage and kitchen-slops for animal food, and in addition to the losses sustained from the immediate effect of such kinds of food, hogs fed upon it are very liable to contract specific infectious diseases such as hog-cholera, swine-plague, and tuberculosis.

5. The enormous amount of loss among garbage-fed hogs, which in this State alone aggregates thousands of dollars annually, suggests the desirability of urging the discontinuing of the practice of collecting swill for such purposes. Certainly if the refuse material is to be used for feeding swine it should be collected and fed while fresh and sweet. When possible, it should be kept dry, and by all means free from alkaline dishwater. It is advisable to cook all kitchen or table refuse before feeding, in order to remove the danger of infection from specific diseases. The only suitable channel for the disposal of dishwater is the sewer.

